# Supplementary material for: 4D spinless topological insulator in a periodic electric circuit
Source: Natl Sci Rev. 2020 Apr 15;7(8):1288–95. doi: 10.1093/nsr/nwaa065 (PMC8289168; doi:10.1093/nsr/nwaa065)
Supplement: nwaa065_Supplemental_File [file nwaa065_supplemental_file.pdf]

Supplementary information for 4D spinless topological insulator in a periodic electric circuit

Rui Yu,[1,*] Y. X. Zhao,[2,3,†] and Andreas P. Schnyder[4]

[1] School of Physics and Technology, Wuhan University, Wuhan 430072, China
[2] National Laboratory of Solid State Microstructures and department of Physics, Nanjing University, Nanjing, 210093, China
[3] Collaborative Innovation Center of Advanced Microstructures, Nanjing University, Nanjing 210093, China
[4] Max-Planck-Institute for Solid State Research, D-70569 Stuttgart, Germany

CONTENTS

The second Chern number 1

Surface Weyl states 2

Circuit systems 3

Simulation details 5

References 6

Appendix A: The second Chern number

The change of second Chern number always accompanies with the gap closing and reopening process, which occurs around the Dirac points. Assuming $f_5 = 0$, there are four Dirac points, located at $D_{1,2} : \pm\frac{2\pi}{3}(-1, 1, -1, 1)$ and $D_{3,4} : \pm\frac{2\pi}{3}(-1, 1, 1, -1)$ in the 4D BZ. Here, we calculate the changes of the second Chern number passing through the first Dirac point, as we tune the mass term in f_5. Expanding $f_i(\boldsymbol{k}), i = 1, 2, 3, 4, 5$ around the Dirac point at $D_1 = \frac{2\pi}{3}(1, -1, 1, -1)$, one obtains $f_1 = -\frac{\sqrt{3}}{2}t(k_1 - k_2)$, $f_2 = -\frac{t}{2}(k_1 + k_2)$, $f_3 = -\frac{\sqrt{3}}{2}t(k_3 - k_4)$, $f_4 = -\frac{t}{2}(k_3 + k_4)$, and $f_5 = m - t$ up to the first order of $\boldsymbol{k}$. Substituting f_i into Eq.(2) in the main text, we get

$$\begin{aligned}
C_2 &= \frac{3}{8\pi^3}\int d^4k \epsilon^{\alpha\beta\gamma\eta\tau}\frac{f_\alpha\partial_{k_1}f_\beta\partial_{k_2}f_\gamma\partial_{k_3}f_\eta\partial_{k_4}f_\tau}{f^5} \\
&= \frac{3}{8\pi^3}\int d^4k\frac{1}{f^5}\begin{vmatrix} f_1 & f_2 & f_3 & f_4 & f_5 \\ \partial_{k_1}f_1 & \partial_{k_1}f_2 & \partial_{k_1}f_3 & \partial_{k_1}f_4 & \partial_{k_1}f_5 \\ \partial_{k_2}f_1 & \partial_{k_2}f_2 & \partial_{k_2}f_3 & \partial_{k_2}f_4 & \partial_{k_2}f_5 \\ \partial_{k_3}f_1 & \partial_{k_3}f_2 & \partial_{k_3}f_3 & \partial_{k_3}f_4 & \partial_{k_3}f_5 \\ \partial_{k_4}f_1 & \partial_{k_4}f_2 & \partial_{k_4}f_3 & \partial_{k_4}f_4 & \partial_{k_4}f_5 \end{vmatrix} \\
&= \frac{9}{32\pi^3}(m-t)t^4\int d^4k\frac{1}{f^5} \\
&= \frac{\text{sgn}(m-t)}{2},
\end{aligned} \tag{S1}$$

where $\text{sgn}(x)$ is the sign of x. The changes of the second Chern number are the same for the Dirac points at D_1 and D_4 due to time-reversal symmetry [1]. With similar calculations, we obtain $C_2 = -\frac{\text{sgn}(2m+t)}{2}$ near the Dirac points $D_{2,3}$. Therefore, the change of the second Chern number should be $\Delta C_2 = -2$ as we increases the mass m across $m = -t/2$, and $\Delta C_2 = 2$ as m increasingly crosses $m = t$. On the other hand, if the mass term tends to negative or positive infinity, it is the only dominant term in the Hamiltonian with the other terms negligible. In these situations, all particles are actually localized in real space on the energy level with energy $-|m|$, and the system behaves like an atomic insulator with topological number $C_2 = 0$. Therefore, we find that the 4D system stays in the topologically nontrivial states with the second Chern number $C_2 = -2$ if $-t/2 < m < t$, as shown in Fig.1***a***.

* yurui@whu.edu.cn
† zhaoyx@nju.edu.cn

Appendix B: Surface Weyl states

We consider the 4D system terminated in the $\boldsymbol{r}_1$ direction with the semi-infinite system in the region of $\boldsymbol{r}_1 > 0$, and investigate the surface states on the $\boldsymbol{r}_1 = 0$ surface. Following the method given in Ref. [2], we write the bulk Hamiltonian (1) in the form,

$$H = \Psi_k^\dagger[\boldsymbol{h}(\boldsymbol{k}) \cdot \boldsymbol{\Gamma}]\Psi_k, . \tag{S1}$$

Here, $\Psi_{\boldsymbol{k}}$ is the vector of quasi-particle annihilation operators, and

$$\begin{aligned}\boldsymbol{h}(\boldsymbol{k}) &= \boldsymbol{b}^0 + \boldsymbol{b}e^{-ik_1} + \boldsymbol{b}^* e^{ik_1} \\ &= \boldsymbol{b}^0 + 2\boldsymbol{b}^r \cos k_1 + 2\boldsymbol{b}^i \sin k_1,\end{aligned} \tag{S2}$$

where $\boldsymbol{b}^0 = (-t(1+\cos k_2), t \sin k_2, f_3, f_4, f_5)$, $\boldsymbol{b}^r = (-t/2, 0, 0, 0, 0)$ and $\boldsymbol{b}^i = (0, t/2, 0, 0, 0)$ are the real and imaginary components of the vector $\boldsymbol{b}$, respectively. $\boldsymbol{b}^0$ can be decomposed into the components $\boldsymbol{b}^0_\perp$ and $\boldsymbol{b}^0_\parallel$, where $\boldsymbol{b}^0_\perp$ is normal to the plane spanned by $(\boldsymbol{b}^r, \boldsymbol{b}^i)$ and $\boldsymbol{b}^0_\parallel$ lies with on the plane. Then we can define

$$\boldsymbol{h}_\parallel(\boldsymbol{k}) = \boldsymbol{b}^0_\parallel + 2\boldsymbol{b}^r \cos k_1 + 2\boldsymbol{b}^i \sin k_1, \tag{S3}$$

as the projection of $\boldsymbol{h}(\boldsymbol{k})$ on the the $(\boldsymbol{b}^r, \boldsymbol{b}^i)$ plane. $\boldsymbol{h}(\boldsymbol{k})$ is on a plane that is offset from the plane containing $\boldsymbol{h}_\parallel(\boldsymbol{k})$ by the vector $\boldsymbol{b}^0_\perp$.

For a fixed parallel momentum $\tilde{\boldsymbol{k}} = (k_2, k_3, k_4)$, $\boldsymbol{h}_\parallel(\boldsymbol{k})$ traces out an ellipse in the $(\boldsymbol{b}^r, \boldsymbol{b}^i)$ plane. As proved in [2], the system has mid-gap edge states if and only if $\boldsymbol{h}_\parallel(\boldsymbol{k})$ encloses the origin. The energy of the edge states is given by the distance of the vector $\boldsymbol{b}^0_\perp$. According to this proof, we obtain that the edge states exist if and only if $0 < 1 + \cos k_2 < 1/2$, which gives that the edge states appears in the 3D BZ with $k_2 \in (2\pi/3, 4\pi/3)$ and $k_{3,4} \in [0, 2\pi]$, with the energies given as $E_{s\pm} = \pm|\boldsymbol{b}^0_\perp|$.

In order to derive the effective Hamiltonian for the surface states, we first define the projector

$$P_\pm = \frac{1}{4}(1 \pm \Gamma^\perp)(1 - i\Gamma^x\Gamma^y), \tag{S4}$$

that projects states onto the surface states with energy $E_{s\pm}$, where $\Gamma^x = \hat{\boldsymbol{v}}_1 \cdot \boldsymbol{\Gamma}$, $\Gamma^y = \hat{\boldsymbol{v}}_2 \cdot \boldsymbol{\Gamma}$ and $\Gamma^\perp = \hat{\boldsymbol{v}}_\perp \cdot \boldsymbol{\Gamma}$. $\hat{\boldsymbol{v}}_1 = (1, 0, 0, 0, 0)$ and $\hat{\boldsymbol{v}}_2 = (0, -1, 0, 0, 0)$ are unit vectors as a coordinate basis of the plane spanned by $(\boldsymbol{b}^r, \boldsymbol{b}^i)$. Therefore the effective surface Hamiltonian is

$$\begin{aligned}H^s &= E_{s+}P_+ + E_{s-}P_- \\ &= |\boldsymbol{b}^0_\perp|\Gamma^\perp \frac{1 + i\Gamma_1\Gamma_2}{2} = \boldsymbol{b}^0_\perp \cdot \Gamma P_s \\ &= (f_1\Gamma_1 + f_2\Gamma_2 + f_5\Gamma_5)P_s\end{aligned} \tag{S5}$$

where $P_s = (1+i\Gamma_1\Gamma_2)/2 = \mathrm{diag}(0, 0, 1, 1)$. Therefore the effective surface Hamiltonian can be written as a two-by-two Hamiltonian

$$H^s_{2\times2} = -f_3\sigma_1 + f_4\sigma_2 + f_5\sigma_3, \tag{S6}$$

with the basis, in terms of the basis of H, $(0, 0, 1, 0)$ and $(0, 0, 0, 1)$. The coefficients $f_{3,4,5}$ are given in the main text, which are functions of $\tilde{\boldsymbol{k}}$. Now it is easy to calculate the Weyl points on the surface. The Weyl points are located at the gap closing points of $H^s_{2\times2}$ as the solutions of the equations $f_3(\tilde{\boldsymbol{k}}) = 0$, $f_4(\tilde{\boldsymbol{k}}) = 0$ and $f_5(\tilde{\boldsymbol{k}}) = 0$ under the constraint $k_2 \in (2\pi/3, 4\pi/3)$. The positions of the two Weyl points are hence given by $2\pi(5/12, 1/3, -1/3)$ and $2\pi(-5/12, -1/3, 1/3)$, which are related by time-reversal symmetry.

To determine the chirality of the Weyl point w_1, we expand the effective surface Hamiltonian around the Weyl point w_1. The Hamiltonian reads $H_{w_1} = \sum_{i,j} k_i a_{ij} \sigma_j$ up to the first order of $\boldsymbol{k}$, where $i, j = 1, 2, 3$ and the matrix a has entries, $a_{11} = 0$, $a_{12} = 0$, $a_{13} = -1$, $a_{21} = \sqrt{3}/2$, $a_{22} = -1/2$, $a_{23} = -1$, $a_{31} = -\sqrt{3}/2$, $a_{32} = -1/2$, $a_{33} = 0$. The chirality χ of the Weyl points is given by the sign of the determinant of matrix a. Because $\chi = \mathrm{sgn}(Det(a)) = +1$ for the w_1 point, we see it is left handed. The Weyl point at w_2 has the same chirality due to the time reversal symmetry.

Appendix C: Circuit systems

In this section, we present the correspondence between the tight-binding lattice model and our proposed circuit lattice. According to the Kirchhoff current law, the currents flowing through the a, b, c, d nodes in the home unit cell of Fig.2 are given as

$$\begin{aligned}
I_a =& \sum_{\boldsymbol{R},\alpha} j\omega C_{a,\boldsymbol{R}_{a\alpha}}(v(\boldsymbol{R}_{a\alpha},\alpha)-v(\boldsymbol{0},a))-v(\boldsymbol{0},a)(j\omega C_{a0}+\frac{1}{j\omega L}),\\
I_b =& \sum_{\boldsymbol{R},\beta} j\omega C_{b,\boldsymbol{R}_{b\beta}}(v(\boldsymbol{R}_{b\beta},\beta)-v(\boldsymbol{0},b))-v(\boldsymbol{0},b)(j\omega C_{b0}+\frac{1}{j\omega L}),\\
I_c =& \sum_{\boldsymbol{R},\gamma} j\omega C_{c,\boldsymbol{R}_{c\gamma}}(v(\boldsymbol{R}_{c\gamma},\gamma)-v(\boldsymbol{0},c))-v(\boldsymbol{0},c)(j\omega C_{c0}+\frac{1}{j\omega L}),\\
I_d =& \sum_{\boldsymbol{R},\delta} j\omega C_{d,\boldsymbol{R}_{d\delta}}(v(\boldsymbol{R}_{d\delta},\delta)-v(\boldsymbol{0},d))-v(\boldsymbol{0},d)(j\omega C_{d0}+\frac{1}{j\omega L}),
\end{aligned} \tag{S1}$$

where I_τ with $\tau = a, b, c, d$ are the currents that flow through node τ, $v(\boldsymbol{0}, \tau)$ is the voltage at node τ in the home unit cell $\boldsymbol{0} = (0, 0, 0, 0)$, $v(\boldsymbol{R}_{a\alpha}, \alpha)$ is the voltage at node α in the unit cell at $\boldsymbol{R}_{a\alpha}$, ω is the resonance frequency of the circuit lattice, $C_{\tau,\boldsymbol{R}_{\tau\alpha}}$ are capacitors connecting nodes τ and α. $C_{\tau 0}$ and L are capacitors and inductors, through which the nodes are connected to ground, with the ground voltage set to zero. Considering the current conservation at each node, namely, the sum of the inflow and outflow currents at every node equal to zero, (S8) can be simplified, and rewritten as

$$\begin{aligned}
(\sum_{\boldsymbol{R},\alpha} C_{a,\boldsymbol{R}_{a\alpha}} + C_{a0})v(\boldsymbol{0},a) - \sum_{\boldsymbol{R},\alpha} C_{a,\boldsymbol{R}_{a\alpha}} v(\boldsymbol{R}_{a\alpha},\alpha) =& \frac{1}{\omega^2 L}v(\boldsymbol{0},a),\\
(\sum_{\boldsymbol{R},\beta} C_{b,\boldsymbol{R}_{b\beta}} + C_{b0})v(\boldsymbol{0},b) - \sum_{\boldsymbol{R},\beta} C_{b,\boldsymbol{R}_{b\beta}} v(\boldsymbol{R}_{b\beta},\beta) =& \frac{1}{\omega^2 L}v(\boldsymbol{0},b),\\
(\sum_{\boldsymbol{R},\gamma} C_{c,\boldsymbol{R}_{c\gamma}} + C_{c0})v(\boldsymbol{0},c) - \sum_{\boldsymbol{R},\gamma} C_{c,\boldsymbol{R}_{c\gamma}} v(\boldsymbol{R}_{c\gamma},\gamma) =& \frac{1}{\omega^2 L}v(\boldsymbol{0},c),\\
(\sum_{\boldsymbol{R},\delta} C_{d,\boldsymbol{R}_{d\delta}} + C_{d0})v(\boldsymbol{0},d) - \sum_{\boldsymbol{R},\delta} C_{d,\boldsymbol{R}_{d\delta}} v(\boldsymbol{R}_{d\delta},\delta) =& \frac{1}{\omega^2 L}v(\boldsymbol{0},d).
\end{aligned} \tag{S2}$$

The current equations for all unit cell can be written in a similar way. Assuming the circuit system has the translational symmetries along the $\boldsymbol{r}_{1,2,3,4}$ directions, the voltage on the nodes satisfies the Bloch theorem, which gives that $v(\boldsymbol{R}_{\tau\alpha} + \boldsymbol{R}, \alpha) = e^{i\boldsymbol{k}\cdot\boldsymbol{R}} v(\boldsymbol{R}_{\tau\alpha}, \alpha)$, where $\boldsymbol{R}$ denotes the lattice index. Performing the Fourier transform, (S9) can be written into a four by four matrix equation in $\boldsymbol{k}$ space. To cast this equation into the same form of the Hamiltonian, Eq.(1), in the main text, we introduced the vectors in (S8) and (S9) as $\boldsymbol{R}_{ad} = \boldsymbol{R}_{bc} \in \{(0,0,0,0),(1,0,0,0),(0,1,0,0)\}$, $\boldsymbol{R}_{ab} = -\boldsymbol{R}_{cd} \in (0,0,0,0),(0,0,1,0),(0,0,0,1)$, $\boldsymbol{R}_{aa} = \boldsymbol{R}_{cc} \in (0,1,1,0),(0,-1,-1,0)$, and $C_{a,\boldsymbol{R}_{ab}} = C_{b,\boldsymbol{R}_{bc}} = C_{a,\boldsymbol{R}_{ad}} = C_{a,\boldsymbol{R}_{aa}} = C_{c,\boldsymbol{R}_{ac}} = -C_{c,\boldsymbol{R}_{cd}} = C$. Exchanging the order of the subscripts, one gets $\boldsymbol{R}_{\alpha\beta} = -\boldsymbol{R}_{\beta\alpha}$ $(\alpha \neq \beta)$ and $C_{\alpha,\boldsymbol{R}_{\alpha\beta}} = C_{\beta,\boldsymbol{R}_{\beta\alpha}}$. Therefore, through straightforward calculations, we can write (S9) as

$$\begin{bmatrix} H_{aa} & H_{ab} & H_{ac} & H_{ad} \\ & H_{bb} & H_{bc} & H_{bd} \\ & & H_{cc} & H_{cd} \\ \dagger & & & H_{dd} \end{bmatrix} \begin{bmatrix} v_a(\boldsymbol{k}) \\ v_b(\boldsymbol{k}) \\ v_c(\boldsymbol{k}) \\ v_d(\boldsymbol{k}) \end{bmatrix} = \frac{1}{\omega^2 L} \begin{bmatrix} v_a(\boldsymbol{k}) \\ v_b(\boldsymbol{k}) \\ v_c(\boldsymbol{k}) \\ v_d(\boldsymbol{k}) \end{bmatrix}, \tag{S3}$$

where $H_{aa} = C_{a0} + 8C - C\cos(k_x + k_2)$, $H_{bb} = C_{b0} + 6C$, $H_{cc} = C_{c0} + 2C - C\cos(k_x + k_2)$, $H_{dd} = C_{d0}$, $H_{ab} = -C(1 + e^{ik_3} + e^{ik_4})$, $H_{ac} = 0$, $H_{ad} = H_{bc} = -C(1 + e^{ik_1} + e^{ik_2})$, $H_{bd} = 0$, $H_{cd} = C(1 + e^{-ik_3} + e^{-ik_4})$, where the parameters of capacitor are choosing as $C_{ab} = C_{bc} = C_{ad} = -C_{cd} = C$, $C_{c0} = C_{a0} + 6C$ and $C_{d0} = C_{b0} + 6C$. The matrix in the left hand side of (S10) can be written in terms of the Γ matrices, with the coefficients given as $f_1(\boldsymbol{k}) = -C(1 + \cos k_1 + \cos k_4)$, $f_2(\boldsymbol{k}) = C(\sin k_1 + \sin k_2)$, $f_3(\boldsymbol{k}) = -C(1 + \cos k_3 + \cos k_4)$, $f_4(\boldsymbol{k}) = C(\sin k_3 + \sin k_4)$, $f_5(\boldsymbol{k}) = \frac{C_{a0} - C_{b0} + 2C}{2} - C\cos(k_2 + k_3)$, $f_0(\boldsymbol{k}) = \frac{C_{a0} + C_{b0} + 14C}{2} - C\cos(k_2 + k_3)$. Comparing with the tight-binding Hamiltonian in the main text, we find the correspondences between the circuit lattice and the tight-binding lattice

model, which are listed below. The capacitance C corresponds to the hopping parameters t with the relation $t = C$, the voltages $(v_a, v_b, v_c, v_d)^T$ correspond to the wave function, and the frequency ω corresponds to the eigenvalues with the relation $\varepsilon = \frac{1}{\omega^2 L}$. The mass term, now characterized by $m = (C_{a0} - C_{b0})/2 + C$, can be tuned by C_{a0} and C_{b0}. As discussed in the main text, the system is topologically nontrivial for $-t/2 < m < t$. We can choose the parameters as C = 1μF, $\mathrm{C_{a0}}$ = 1C, $\mathrm{C_{b0}}$ = 3C, $\mathrm{C_{c0}}$ = 7C, $\mathrm{C_{d0}}$ = 9C, R = 1MΩ, and L = 1μH, with $C_{b0} = C_{a0} + 2C$, which lead to that the case of $m = 0$ corresponds to the second Chern number $C_2 = -2$.

We now address how to construct the 4D circuit lattice on a 2D circuit board. We first consider the connections in the ($\boldsymbol{r}_3$,$\boldsymbol{r}_4$) space. The capacitors connecting the nodes separated by the vectors, R_{ab} and R_{cd}, are placed on this plane. Choosing the Born-von Karman periodic boundary conditions (PBCs) for the circuit lattice, the voltage on the nodes satisfies $v(\boldsymbol{R} + N_i \boldsymbol{r}_i, \alpha) = v(\boldsymbol{R}, \alpha)$, where $\boldsymbol{R}$ is the lattice index, α is the node index, and i runs over the dimensions of the 4D space. With the PBCs in the $\boldsymbol{r}_3$-$\boldsymbol{r}_4$ space, the a, d nodes on the right (top) edge are connected to the b, c nodes on the left (bottom) edge, and the electronic devices are placed on a finite block with cyan color as shown in Fig.2 **a**. For the convenience of the connections in the $\boldsymbol{r}_1$ and $\boldsymbol{r}_2$ directions, we connect all nodes to the edge of the block by wires. The small black solid squares with the same label denote they are equipotential points connected by wires. The wires are not shown in Fig.2 **a** to make the figures neat. Recalling that the hopping parameters t_{cd} have opposite signs comparing to other hopping parameters, which leads to the capacitors connecting c-d nodes have opposite signs compared to other capacitors. Here we choose the capacitors C_{cd} with negative capacitance. In Fig.2 **a**, the positive capacitors are indicated by red solid line while the negative ones are indicated by red dashed lines. The sub-circuit to realize the negative capacitor is given in Fig.2 **d**, which will be proved later. With the circuit block given in Fig.2 **a**, the connections on the $\boldsymbol{r}_1$-$\boldsymbol{r}_2$ plane are schematically shown in Fig.2 **e**. The capacitors, connecting the a-d, b-c, a-a, and c-c nodes that are separated by R_{ad}, R_{bc}, R_{aa}, and R_{cc}, are indicated with red solid line. The black lines indicate the wires and the the small blue square at the lines crossing point means these lines are connected, lines with no square mean that they are not connected. Experimentally, we can use the multi-layer board and put the lines on different layers to avoid the connection between them.

Capacitors on the circuit lattice connecting nodes c-d are chosen to have negative capacitance. Here we design a two-port device, whose I-V property behaves as a capacitor with negative capacitance. The circuit diagram is shown in Fig.S1 **a**, where the operation amplifiers, indicated by the triangle shape, are used. For an ideal operational amplifier, there is no voltage across its inputs. Therefore the input terminals V_+ and V_- behave like a short circuit. But this kind of short is virtual, different from a real one, and draws no current because of the infinite impedance between the two inputs. We assume the voltage at A and B nodes are V_2 and V_1, respectively. The voltage on the other nodes are marked as shown in Fig.S1 **a**. Some nodes have the same voltage as A or B nodes for the virtual short property between +, − nodes of the operation amplifiers. Then according to the Kirchhoff's current law, we obtain the current outflow for the A nodes and inflow for the B nodes, which are explicitly given as

$$\begin{aligned} i_1 =& j\omega C(V_{o2} - V_1), \\ i_2 =& j\omega C(V_2 - V_{o1}). \end{aligned} \tag{S4}$$

For the currents in the internal branch, we get

$$\begin{aligned} (V_{o1} - V_2)/R =& (V_2 - V_1)/R, \\ (V_1 - V_{o2})/R =& (V_2 - V_1)/R. \end{aligned} \tag{S5}$$

which lead to $V_2 - V_{o1} = -(V_2 - V_1)$ and $V_{o2} - V_1 = -(V_2 - V_1)$. Substituting these identities in to eqs.(S11), we have

$$i_1 = i_2 = -j\omega C(V_2 - V_1). \tag{S6}$$

Comparing to a positive valued capacitor, here the I-V relation satisfies $I/V = j\omega C$. Therefore the above result shows that the two-port device given in Fig.S1 **a**, whose I-V properties behaves effectively as a capacitor with negative capacitance $-C$.

The circuit in Fig.S1 **b** can be simplified as given in Fig.S1 **c**, where two operational, OP_3 and OP_4, working as a voltage follower are removed. In this case, we have

$$\begin{aligned} i_{11} =& j\omega C(V_2 - V_{o1}), \\ i_{12} =& (V_2 - V_1)/R, \\ i_{21} =& (V_2 - V_1)/R, \\ i_{22} =& j\omega C(V_{o2} - V_1). \end{aligned} \tag{S7}$$

The currents in the internal branch satisfy the relations given in (S12). With above equations we get

$$
\begin{aligned}
I_1 =& i_{11} + i_{12} = (V_2 - V_1)(\frac{1}{R} - j\omega C),\\
I_2 =& i_{21} + i_{22} = (V_2 - V_1)(\frac{1}{R} - j\omega C).
\end{aligned} \tag{S8}
$$

Therefore, if R is large enough, we have $I_1 = I_2 \approx -j\omega C(V_2 - V_1)$, getting the same result as given in (S13). The simulation result for the circuit in Fig.S1 **a** is shown in Fig.S1 **c**, which is consistent with the analytic result.

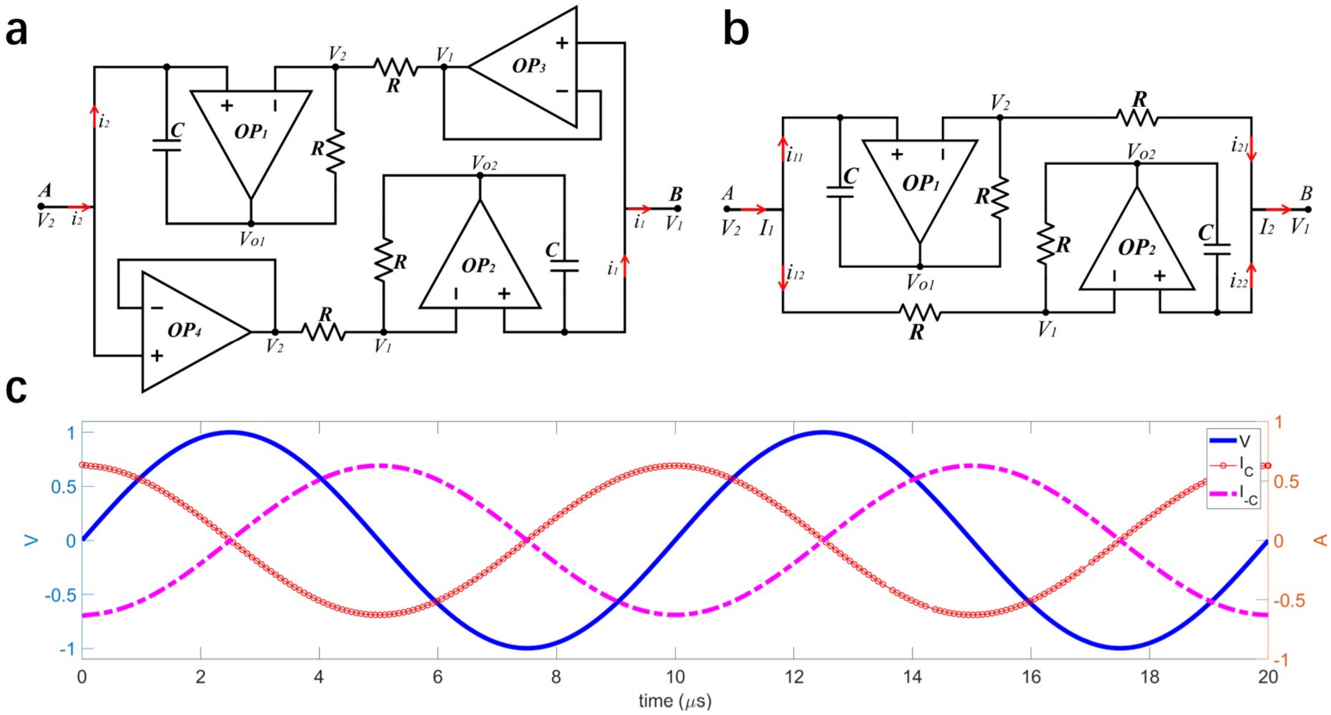

FIG. S1. The designed capacitor with effective capacitance $-C$. **a**, The sub-circuit to realize the $-C$ capacitor. Four operational amplifiers are used, where the OP_3 and OP_4 are used as voltage follower. **b**, The same as **a**, but with OP_3 and OP_4 being removed. If R is sufficiently large, this circuit is approximately equivalent to the circuit in **a**. **c**, The simulation results for the circuit in **a**. The voltage at node A are plotted by the blue solid line and the current flow out node A is plotted by the red circle line . The pink dashed line denotes the current for a $+C$ capacitor connected to the same voltage source. From the simulated V-I properties of the circuit, the sub-circuit behaves effectively as a capacitor with negative capacitance, which is consistent with the analytic results.

Appendix D: Simulation details

We perform the simulations of the circuit lattice by using the LTspice software. In order to obtain the resonance frequency of the circuit lattice, we probe the voltage $v(t, \boldsymbol{R}, \alpha)$ at each node as a function of time, and then extract the frequency information from $v(\omega, \boldsymbol{k}, \alpha)$, which can be obtained by the Fourier transform of $v(t, \boldsymbol{R}, \alpha)$. To excite the voltage signals at each node, we use a pulse source, which is connected at the four nodes in the (1,1,1,1) unit cell, and is then removed immediately after the voltage at each nodes starting to oscillate. Then we collect the voltage

data $v(t, \boldsymbol{R}, \alpha)$. The parameters of the pulse source are set as $pulse(V_{initial} = 0, V_{on} = 1V, V_{delay} = 0us, V_{rise} = 1us, V_{fall} = 1us, T_{on} = 1us, T_{period} = 4us)$, where $V_{initial}$ is the voltage when the pulse is not on, V_{on} is the value when the pulse is fully turned on, T_{delay} is the time delay, T_{rise} is the rise time of the pulse, T_{fall} is the fall time of the pulse, T_{on} is the pulse width, and T_{period} is the period of the pulse. The ideal operational amplifier are used in the sub-circuit for negative value capacitors. The pulse voltage source are connected at the four nodes in the $(1, 1, 1, 1)$ lattice and removed after $100\mu s$ when the whole system is activated. The parameters for transient analysis are set as $tran(t_{step} = 5\mu s, t_{stop} = 2ms)$, which saves the voltage at every node every $5\mu s$ to $2ms$. After the $v(t, \mathbf{R}, \alpha)$ data are obtained, we can extract the band dispersion as discussed in the main text .

Here, we provide the details for the open boundary conditions in the $\boldsymbol{r}_1$ direction as discussed in the main text. As shown in Fig.2 **e**, we remove the capacitors that are used to connect node-a (b) on the top edge and node-d (c) on the bottom edge. Recalling that the onsite energy of a node corresponds to the sum of all the capacitors connected to this node, the removal of the capacitors on the top (bottom) edge changes the onsite energy of node-a, b (and c, d) on the top (bottom) edge. In order to ensure that our simulation results can be compared with those obtained from the tight-binding model, extra capacitors with capacitance C is used to connect these four types of removal nodes to ground, respectively, to make the onsite energy of these nodes equal that of the bulk nodes. If we remove the capacitors that connecting the a (b) nodes on the top edge and d (c) nodes on the bottom edge but not repair these two edges by adding capacitors as we did above, the surface onsite energy on these nodes will be different from that of bulk nodes. The changes of surface onsite energy can just shift the position of Weyl points in momentum and frequency space, rather than make them disappear, since the Weyl points can disappear only when Weyl points with opposite topological charges are annihilated in pairs. In the 4D topological system, the surface Weyl points with the same topological charges are located on one surface, while the opposite topological charged Weyl points are separated spatially on the other surface. Therefore the 4D surface Weyl points are stable.

[1] B. A. Bernevig and T. L. Hughes, *Topological insulators and topological superconductors* (Princeton University Press, Princeton, New Jersey, 2013).

[2] Roger S. K. Mong and Vasudha Shivamoggi, "Edge states and the bulk-boundary correspondence in Dirac hamiltonians," Phys. Rev. B **83**, 125109 (2011).
